# A Four-Gene Signature Associated with Radioresistance in Head and Neck Squamous Cell Carcinoma Identified by Text Mining and Data Analysis

**DOI:** 10.1155/2022/5693806

**Published:** 2022-09-27

**Authors:** Yongqian Zhang, Hongmin Wang, Feifei Wang, Wenhua Ma, Na Li, Changwen Bo, YingChun Zhao, Li He, Ming Liu

**Affiliations:** ^1^Department of Oncology, The First Hospital of Hebei Medical University, Shijiazhuang, 050031 Hebei, China; ^2^Department of Neurology, The First Hospital of Hebei Medical University, Shijiazhuang, 050031 Hebei, China; ^3^Department of Radiotherapy and Oncology, The Third Hospital of Hebei Medical University, No. 139, Ziqiang Road, Shijiazhuang, 050051 Hebei, China

## Abstract

**Purpose:**

Head and neck squamous cell carcinoma (HNSCC) is the sixth leading cancer globally, and radiotherapy plays a crucial part in its treatment. This study was designed to identify potential genes related to radiation resistance in HNSCC.

**Method:**

We first used text mining to obtain common genes related to radiotherapy resistance and HNSCC in published articles. Functional enrichment analyses were conducted to identify the significantly enriched pathways and genes. Protein and protein interactions were performed, and the most significant gene modules were determined; then, genes in the gene modules were validated at transcriptional levels and overall survival. Gene set variation analysis (GSVA) score was calculated, and the association between GSVA score and survival/pathway was estimated. Immune cell infiltration, methylation, and genetic alteration analysis of these genes was conducted in HNSCC patients. Finally, potential sensitive anticancer drugs related to target genes were obtained.

**Result:**

We identified 583 common genes through text mining. After further validation, a four-gene signature (EPHB2, SPP1, SERPINE1, and VEGFC) was constructed. The patients with higher GSVA scores have a worse prognosis than those with lower GSVA scores. Differences in methylation of these four genes in HNSCC tumor tissue and normal tissue were compared, with higher methylation levels of EBPH2 and SPP1 in normal tissue and higher methylation levels of SERPINE1 in the tumor. Immune cell infiltration revealed that the increased expression of these genes was closely related to the infiltration level of CD4+ T cell, neutrophil, macrophage, and dendritic cell. Thirty drugs, including 22 positively and eight negatively correlated drugs that most correlated with related genes, were available for treating HNSCC.

**Conclusion:**

In this study, we identified four potential genes as well as corresponding drugs that might be related to radioresistance in HNSCC patients. These candidate genes may provide a promising avenue to further elevate radiotherapy efficacy.

## 1. Introduction

Head and neck squamous cell carcinoma (HNSCC) is the sixth most common type of cancer diagnosed in the world, with 890,000 new cases and 450,000 deaths annually [1, 2], and the incidence increases year by year [3]. HNSCC originates from mucosal epithelium cells in the oral cavity, oropharynx, hypopharynx, and larynx, accounting for 90% of all head and neck cancer [4]. The main risk factors of HNSCC include smoking, heavy drinking, exposure to environmental pollutants, and infection with human papillomavirus and EB virus.

The treatment of HNSCC differs according to the stage of disease, anatomical site, and surgical accessibility, including surgery, radiotherapy, systemic chemotherapy, targeting, and immunotherapy. As a noninvasive and function-preserving therapy, radiation therapy plays a crucial role in curative-intent treatments for head and neck cancers. It can be used as a single treatment for early tumors or combined with surgery or concurrent chemotherapy as a treatment for advanced tumors [5].

Although there has been tremendous progress in treatment in recent years, the survival rate of head and neck cancer has not been significantly improved [6, 7]. The efficacy of radiotherapy is affected by the sensitivity of radiotherapy, and radiotherapy resistance would lead to treatment failure. A mechanism for radiotherapy resistance is hypoxia. Adequate oxygen can maintain the sensitivity of tumor tissue to radiotherapy, and hypoxia can lead to radiotherapy resistance, thereby reducing the effect of radiotherapy. In addition to hypoxia, there are several molecular and biological mechanisms of radiation resistance, including alterations in intracellular pathways involved in DNA damage and repair, apoptosis, proliferation, and angiogenesis [8]. A couple of signaling pathways related to radiotherapy resistance have been intensively studied, and the three most clinically relevant pathways are EGFR, PI3K/Akt/mTOR, and P53 [9].

Identifying potential genes and pathways associated with radiotherapy resistance may facilitate proper treatment selection and improve outcomes. With the development of bioinformatics, text mining and data analysis have been applied to several aspects of cancer research, such as identifying potential key gene targets, signaling pathways, and predicting the outcomes [10, 11].

**Figure 1:**
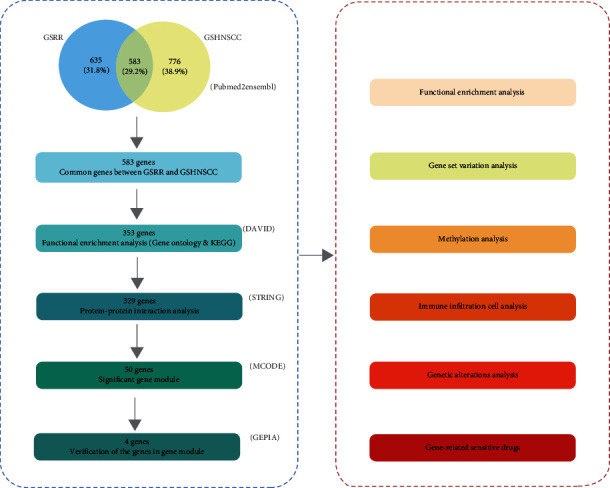
The workflow of the study. GSRR: gene set of radioresistance; GSHNSCC: gene set of head and neck squamous cell carcinoma.

In this study, we first used text mining to obtain common genes, which represent the genes related to radiotherapy resistance and HNSCC in published articles. Secondly, a four-gene signature associated with radioresistance was identified, and the roles of the gene signature in the prognosis, pathway, methylation, immunity, and genetic alterations of HNSCC were explored. Finally, multiple potential targeted drugs associated with radioresistance were established for HNSCC patients.  Figure 1 shows the workflow of this study.

## 2. Materials and Methods

### 2.1. Text Mining

The database pubmed2ensembl was used to perform text mining. pubmed2ensembl provides links between literature and genes, which can be used for data mining. When a query is executed, it extracts all genes related to the search keywords from available biological literature [12]. Firstly, we inputted the keywords “radioresistance,” “radiation resistance,” and “radiotherapy resistance” into the website, respectively, and then, the system extracted all related genes. After removing the duplicate genes, these gene sets made up the gene set of radioresistance (GSRR). Secondly, we performed a query using the keyword “head and neck squamous cell carcinoma,” and the gene sets of head and neck squamous cell carcinoma (GSHNSCC) were obtained. The intersection of GSRR and GSHNSCC was common genes and then used for further analysis.

### 2.2. Functional and Signal Pathway Enrichment Analysis

GO provides a controlled glossary of terms to clarify the characteristics of a gene product through their annotations. The GO terms reflect what is currently known about a gene in terms of biological process (BP), cellular component (CC), and molecular function (MF) [13, 14]. Furthermore, the Kyoto Encyclopedia of Genes and Genomes (KEGG) [15] provides data sources of known biological pathways to mark a gene or group of genes/proteins with their respective KEGG pathways. The GO and KEGG enrichment analysis of common genes was conducted using an online tool DAVID [16], a functional annotation and bioinformatics microarray analysis website. FDR (false discovery rate) < 0.05 was considered statistically significance.

Genes from the significant enrichment terms for BP, CC, MF, and KEGG signal pathways were downloaded from the website DAVID, extracted using R software, and used in the following analysis.

### 2.3. Protein-Protein Interaction Analysis and Gene Module Analysis

The database STRING was used to display the protein-protein interaction (PPI) network of the screened genes. There are seven ways in which proteins interact with each other built into the database, including text mining, experiments, databases, coexpression, cooccurrence, neighborhood, and gene fusion. The genes selected from the GO and pathway enrichment analysis were used as the input set. The minimum required interaction score was set to 0.90 (highest confidence) to further narrow the candidate gene field. The interaction file was downloaded in TSV format and processed using Cytoscape software. The plugin Molecular Complex Detection (MCODE) and STRING in Cytoscape were used to extract significant gene modules in the PPI network. The parameter settings were default values, except the *K*-core was set to 9. Then, the two most significant gene modules, including 50 genes, in the PPI network were selected for further analysis.

### 2.4. Verification of the Genes in TCGA/GTEx

To screen for specific genes, we verified the gene expression levels and OS of these 50 genes on the webserver GEPIA, which could deliver fast and customizable functionalities based on The Cancer Genome Atlas (TCGA) and Genotype-Tissue Expression (GTEx) data [17]. As for gene expression validation involved in 519 HNSCC tumor samples and 44 normal samples, the threshold with ∣log_2_ FC | ≥1 and *P* value < 0.01 was considered statistically significant. For overall survival (OS) analysis of the HNSCC dataset, 362 patients with available OS time data were classified into the low-expression and high-expression groups according to the median transcripts per kilobase million (TPM), and log-rank *P* < 0.05 was considered to be significant.

### 2.5. Gene Set Variation Analysis

To further observe the performance of these four genes as a whole in various aspects, the gene set variation analysis (GSVA) [18] score, which represents the integrated level of the expression of a gene set, was calculated through the R package GSVA. Moreover, the association between GSVA score and survival/pathway/methylation in HNSCC was estimated using the online website GSCA [19].

### 2.6. Immune Cell Infiltration Analysis

The composition and abundance of immune cells in the tumor microenvironment occupy a crucial role in tumor progression and the efficacy of immunotherapy. The website Tumor Immune Estimation Resource [20] (TIMER, https://cistrome.shinyapps.io/timer/) was utilized to further explore the potential association of gene expression and immune infiltration levels of immune cells, including B cell, CD8+ T cell, CD4+ T cell, macrophage, neutrophil, and dendritic cell (DC). *P* < 0.05 was considered statistically significant.

### 2.7. Genetic Alteration Analysis

We further investigated the genetic alterations of these four genes at the DNA level using the cBioPortal. cBioPortal [21] (http://www.cbioportal.org) is a comprehensive online tool providing us with visual and multidimensional cancer genomics data based on TCGA database.

### 2.8. Drug Sensitivity and Gene Expression

To explore potential drugs that can regulate the sensitivity of radiotherapy, we used the online website GSCA to further seek sensitive drugs related to target genes. The database contains the IC50 of 265 small molecules in 860 cell lines and its corresponding mRNA gene expression from Genomics of Drug Sensitivity in Cancer (GDSC) [19]. These drug candidates that target genes associated with radiotherapy resistance may herald the arrival of new potential therapies. Materials and Methods should contain sufficient detail so that all procedures can be repeated. It may be divided into headed subsections if several methods are described.

## 3. Results

### 3.1. Text Mining

Based on the text mining strategy described in Methods, after deleting the duplicates, 1218 genes were found in GSRR, 1359 genes were found in GSHNSCC, and 583 genes were common to both lists.

### 3.2. Functional and Signal Pathway Enrichment Analysis

**Figure 2:**
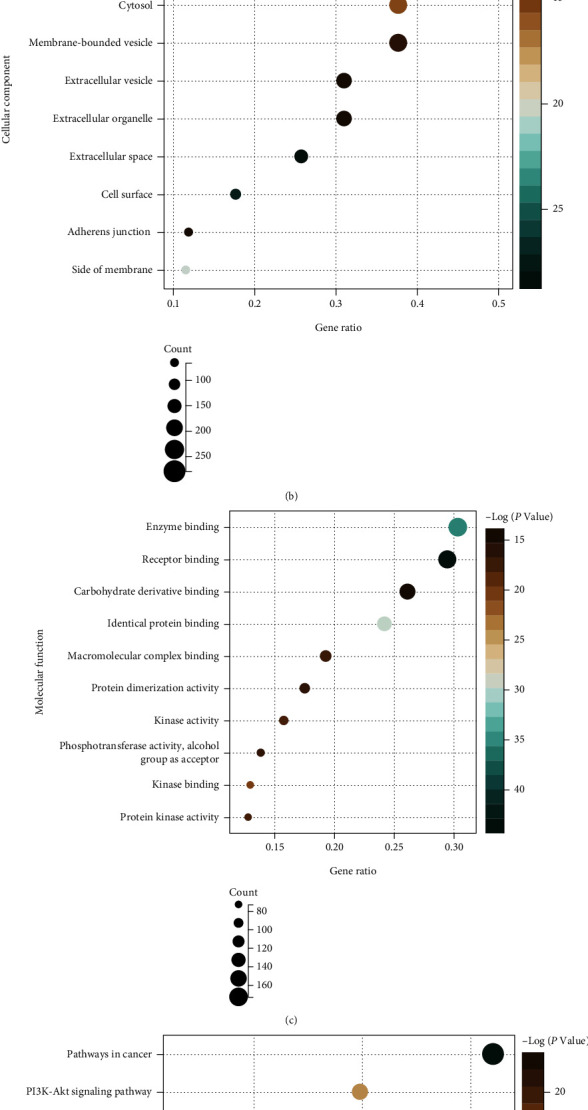
Functional enrichment of common genes. (a–c) The top ten significant GO terms of common genes. (d) The top ten significant KEGG pathways of common genes.

The common genes were uploaded to the DAVID website to identify the GO and pathway terms. Figures 2(a)–2(d) show the top 10 significant enrichment terms for BP, CC, MF, and KEGG signal pathways. In BP annotation, it was mainly involved in response to organic substance, positive regulation of metabolic process, and cellular response to chemical stimulus (Figure 2(a)). In CC annotation, it was significantly involved in the extracellular region, cytosol, and membrane-bounded vesicle (Figure 2(b)). In MF annotation, it was mainly enriched in enzyme binding, receptor binding, and carbohydrate derivative binding (Figure 2(c)). As for signal pathway enrichment, it was primarily involved in the pathways in cancer, PI3K-Akt signaling pathway, and HTLV-I infection (Figure 2(d)), respectively. We extracted the genes that were all significantly enriched in BP, CC, MF, and KEGG signaling pathways, and there were 353 common genes left for the subsequent analysis.

### 3.3. Protein-Protein Interaction Analysis and Gene Module Analysis

The screened genes were entered into the STRING website, and the TSV file was obtained, then downloaded, and analyzed using Cytoscape software.

**Figure 3:**
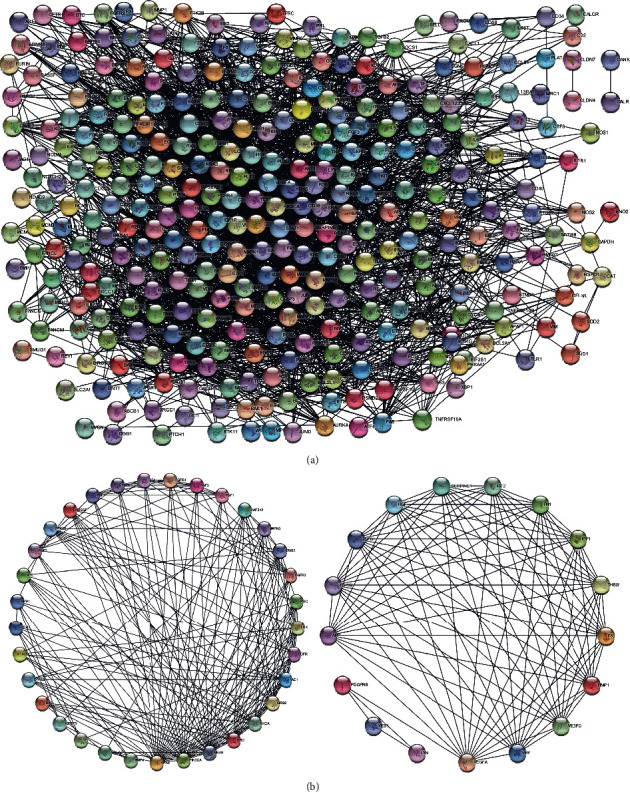
The construction of protein-protein interaction (PPI) networks and significant gene module analysis. (a) The entire PPI networks of common genes. (b) The two most significant gene modules in the PPI networks.

The obtained PPI networks consisted of 329 genes/nodes and 3068 edges (Figure 3(a)), and 24 genes were not involved in the PPI networks. Then, the two most significant gene modules were clustered via MCODE APP built-in Cytoscape. Module 1 consisted of 33 genes/nodes and 191 edges, while module 2 was made up of 17 genes/nodes and 95 edges (Figure 3(b)).

### 3.4. Verification of the Genes in TCGA/GTEx

**Figure 4:**
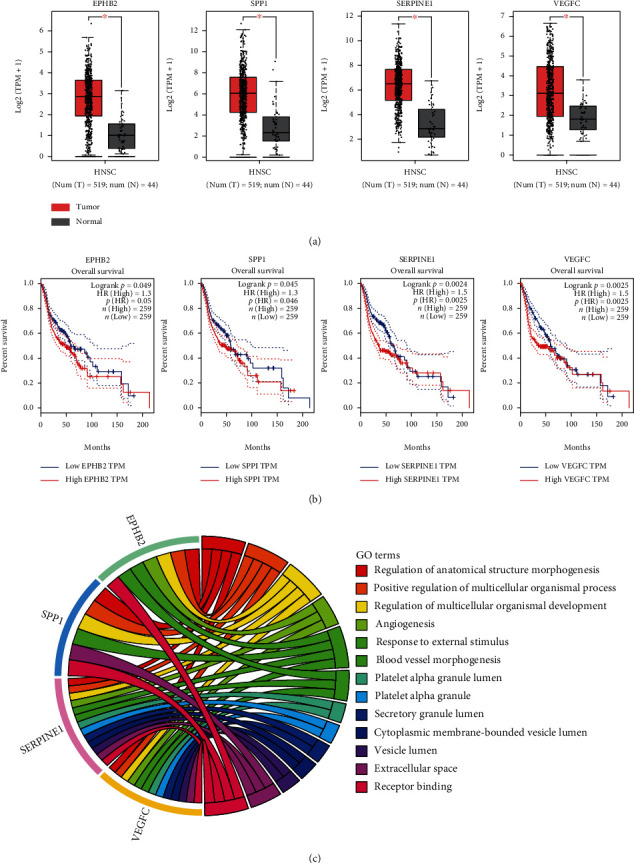
Verification of the filtered four genes and their functional annotation. (a) Validation of the gene expression of EPHB2, SPP1, SERPINE1, and VEGFC in HNSC datasets. The cutoff: ∣log_2_ fold change (FC) | ≥1, and *P* < 0.01 (∗ indicates *P* < 0.01). (b) Overall survival analysis of EPHB2, SPP1, SERPINE1, and VEGFC in HNSC datasets. (c) Chord plot for functional enrichments of four genes.

We verified the expression between normal and cancerous tissues and the survival between high and low expression of these 50 genes in GEPIA (Figures S1–S4). Finally, four genes were filtered (EPHB2, SPP1, SERPINE1, and VEGFC). Their expression in tumor tissues was significantly higher than in normal tissues (Figure 4(a)), and the OS of high expression is worse than low expression (Figure 4(b)). Moreover, the functional annotation of these four genes was conducted using DAVID. Enrichment analysis showed that these four candidate genes are significantly enriched in regulation of anatomical structure morphogenesis (BP, *P* = 2.51*E* − 04), platelet alpha granule lumen (CC, *P* = 0.011), and receptor binding (MF, *P* = 8.41*E* − 04), respectively (Figure 4(c), Table 1).

### 3.5. Gene Set Variation Analysis

**Figure 5:**
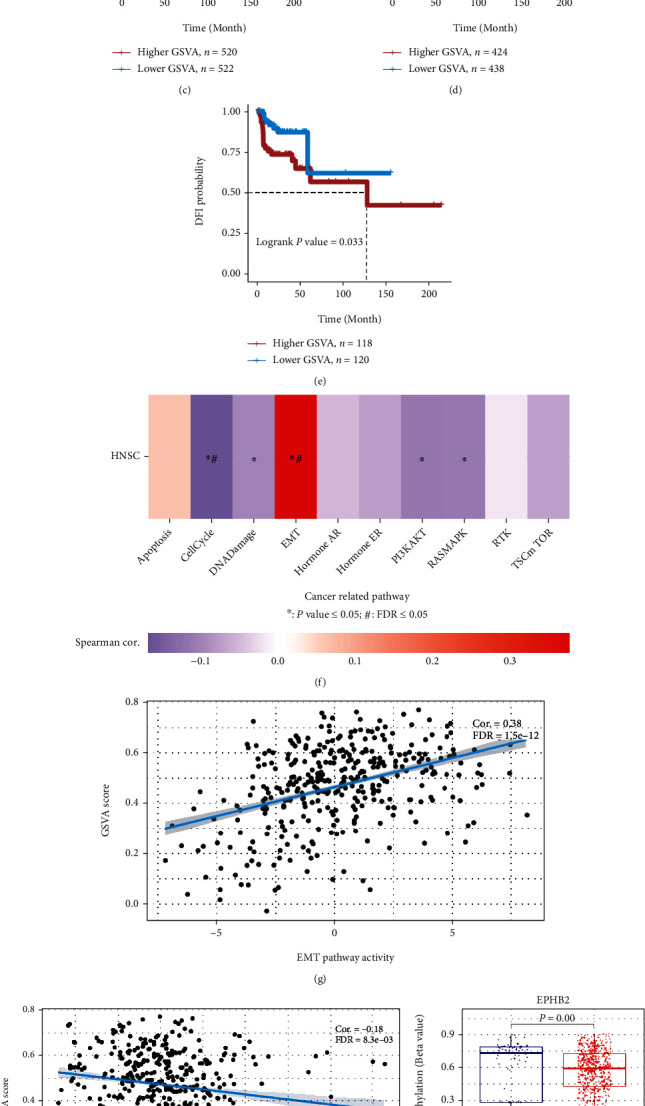
GSVA and methylation analysis. (a) Boxplot showing the GSVA score of the tumor and normal tissue groups. (b–e) OS, PFS, DFS, and DFI analysis between higher and lower GSVA score in HSCC patients. (f) Overview of the relationship between GSVA score and activity of tumor-related pathways. (g) Correlation between GSVA score and EMT pathway activity. (h) Correlation between GSVA score and cell cycle pathway activity. (i–k) Methylation analysis of EPHB2, SPP1, and SERPINE1.

GSVA score was calculated and compared between tumor and normal samples in HNSCC samples on the GSCA. The GSVA score of the tumor group was significantly higher than that of the normal tissue group (Figure 5(a)). Furthermore, the association between GSVA score and survival (includes overall survival (OS), progression-free survival (PFS), disease-specific survival (DSS), and disease-free interval (DFI)) was estimated, and it was found that the GSVA score had a significant relationship with OS, PFS, DSS, and DFI in HNSCC patients (Figures 5(b)–5(e)). These findings suggest that this 4-gene signature has a high potential for predicting poor prognosis in HNSCC patients by assessing their risk score based on related gene expression levels.

Figure 5(f) shows the correlation between GSVA score and activity of cancer-related pathways in HNSCC. These four genes were positively correlated with EMT pathway activity (*P* value = 0.00, FDR = 1.5*e* − 12, Figure 5(g)) and negatively associated with cell cycle pathway activity (*P* value = 0.00, FDR = 8.3*e* − 03, Figure 5(h)).

### 3.6. Methylation Analysis

Differences in methylation of these four genes in HNSCC tumor tissue and normal tissue were compared (Figures 5(i)–5(k)), with higher methylation levels of EBPH2 and SPP1 in normal tissue and higher methylation levels of SERPINE1 in tumor (*P* < 0.05). There is no difference in methylation levels of VEGFC between the two groups.

### 3.7. Immune Infiltration Cell Analysis

**Figure 6:**
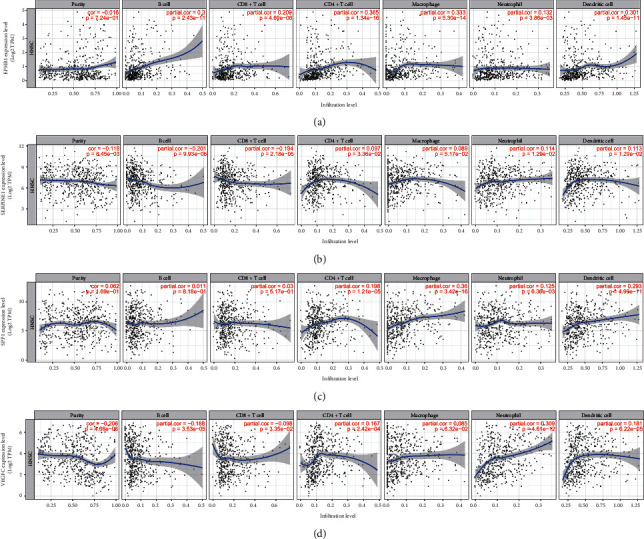
Association of gene expression and immune infiltration levels of immune cells including CD4+ T cell, CD8+ T cell, macrophage, dendritic cell, neutrophil, and B cell.

We used the TIMER database to explore the association between the expression of the selected genes and immune infiltrating cells, including B cell, CD8+ T cell, CD4+ T cell, macrophage, neutrophil, and dendritic cell (DC) in HNSCC. The result revealed that the expression of EPHB2 was positively correlated with all of these immune cells' expression levels. The expression of SPP1 was positively correlated with the expression levels of CD4+ T cell, macrophage, neutrophil, and DC. The expression of SERPINE1 was positively correlated with the expression levels of CD4+ T cell, neutrophil, and DC, while negatively associated with B cell and CD8+ T cell. The expression of VEGFC was positively correlated with CD4+ T cell, neutrophil, and DC, while negatively correlated with B cell and CD8+ T cell (Figures 6(a)–6(d)).

### 3.8. Genetic Alteration Analysis

**Figure 7:**
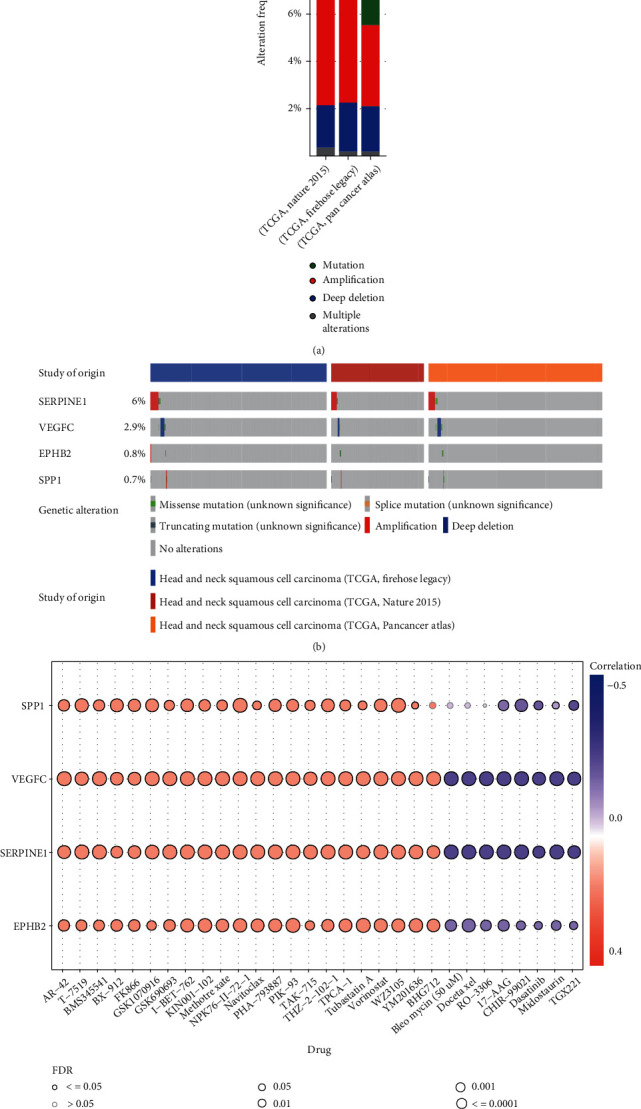
Genetic alteration analysis and exploration of targeted drugs. (a, b) Genetic alteration analysis of the four genes in three HNSCC datasets. (c) Correlation between GDSC drug sensitivity and gene expression.

At the cBioPortal, we examined the genetic alterations of these four prognostic genes in 3 HNSCC studies from TCGA. Overall, we found that these genes were altered in 126 (9%) of all case, with 30 (10.75%) of 279 cases (TCGA, Nature 2015), 50 (9.43%) of 530 cases (TCGA, Firehose Legacy), and 46 (8.8%) of 523 cases (TCGA, PanCancer Atlas), respectively (Figure 7(a)). The amplification of SERPINE1 and the deep deletion of VEGFC were the most frequent copy number alterations (CNA) among these genes (Figure 7(b)).

### 3.9. Discovery of Gene-Related Sensitive Drugs

The four genes were used for drug-sensitivity analysis on the website GSCA.  Figure 7(c) summarizes the top 30 drugs most associated with gene expression in pan-cancer. The drugs are ranked by the integrated level of correlation coefficient and FDR of searched genes, including 22 positively correlated drugs and eight negatively correlated drugs. Information on these sensitive drugs may provide new ideas for further alleviating radioresistance. Results and Discussion may be presented separately, or in one combined section, and may optionally be divided into headed subsections.

## 4. Discussion

HNSCC is one of the most common malignant tumors that seriously threaten human health, and radiotherapy plays a crucial part in its treatment. Radiotherapy resistance can reduce the local efficacy of HNSCC patients and lead to tumor recurrence and distant metastasis and is significantly associated with poor prognosis. Identifying new genes related to radiation resistance in HNSCC may further improve the prognosis of these patients. In this study, we adopted the approach of text mining and bioinformatics, and finally, four potential genes (EPHB2, SPP1, SERPINE1, and VEGFC) were found.

The erythropoietin-producing hepatocellular carcinoma receptor B2 (EPHB2), an essential member of the erythropoietin-producing hepatocellular carcinoma (EPH) receptor family, has been confirmed to be highly expressed in various tumors, such as breast cancer, meningioma, colorectal cancer, gastric cancer, and lung cancer [22]. High expression of EPHB2 is considered a proto-oncogene by promoting blood vessel formation by enhancing cancer cell motility, invasion, and metastasis. Thus, the EPHB2 gene is regarded as a pro-oncogene. EPHB2 is found to play a critical role in the progression from early-stage cutaneous squamous cell carcinoma to an advanced stage, and it is speculated to be a therapeutic target for invasive cutaneous squamous cell carcinoma [23]. Another study has demonstrated that EPHB2 expression regulates angiogenesis both in vitro and in vivo and that EPHB2 overexpression is associated with poor prognosis and tumor angiogenesis in HNSCC patients [24]. Bhatia et al. observed the expression of EPHB2 in the myeloblastoma cell line, and the cell line was more sensitive to radiotherapy after the EphB2 was knocked out. The radiosensitization effect was partly mediated by enhancing G2/M cell cycle arrest. It suggests that blocking the EPHB2 receptor may be a way to improve radiation sensitivity in medulloblastoma [25].

Secreted phosphoprotein 1 (SPP1), also known as osteopontin (OPN), is an extracellular matrix protein closely associated with malignant tumors [26]. It is highly expressed in breast cancer [27], non-small-cell lung cancer [28], prostate cancer [29], and liver cancer [30]. It is associated with tumor invasion and metastasis, apoptosis inhibition, angiogenesis, and chemotherapy resistance [31]. The level of SPP1 mRNA in HNSCC was higher than that in noncancerous tissues, and high SPP1 mRNA level was associated with poor overall survival of HNSCC patients [32]. Another study showed that the serum SPP1 level of HNSCC with positive cervical lymph nodes was significantly higher than that with negative cervical lymph nodes, and researchers concluded that SPP1 might play an essential role in developing cervical lymph nodes metastasis in NHSCC patients [33].

Serpin family E member 1 (SERPINE1), also named plasminogen activator inhibitor type 1 (PAI-1), is a fibrinolysis inhibitor found to be abnormally expressed in gastric cancer [34], colon cancer [35], diffuse lower-grade gliomas [36], and other cancers. It is a biomarker of the poor prognosis of the malignant tumor. Overexpression of SERPINE1 promotes tumor progression, lymph node metastasis, and lung metastasis of HNSCC and is associated with poor prognosis [37]. Yang et al. found that SERPINE1 was significantly overexpressed in HNSCC and correlated with the prognosis of HNSCC patients [38]. Wang et al. constructed a 6-gene signature (including SERPINE1) that can be used to predict the survival of HNSCC patients [39]. Similar to our findings, Lee et al. found that SERPINE1 was associated with radioresistance in HNSCC, and knockdown of SERPINE1 increased radiosensitivity in head and neck cancer tumor-initiating cells [40].

Vascular endothelial growth factor-C (VEGFC) is one of the most crucial lymphangiogenic growth factors, which can stimulate the formation of tumor lymphatics [41]. Recent evidence suggests that VEGFC is also an immunomodulator that modulates the immune system, making tumor cells more likely to evade immune surveillance [42]. Tacconi et al. demonstrated that VEGFC could accelerate tumor growth via fostering cancer immune escape [43]. High expression of VEGFC is significantly associated with poor prognosis in various malignancies, including lung cancer [44], gastric cancer [45], thyroid cancer [46], colorectal cancer [47], and HNSCC [48]. Furthermore, a study showed that silencing VEGFC can increase the radiosensitivity of nasopharyngeal carcinoma CNE-2 cells [49].

Previous studies on radiosensitivity in head and neck cancer have identified some related genes. Some are based on the gene expression levels of cell lines [50, 51], and some are based on gene expression levels in tumors and patient survival data [52, 53]. Foy et al. analyzed the data from the public database by data mining. They found that 13 genes were associated with *in vitro* and *in vivo* radioresistance for HPV-negative HNSCC [54]. Although these genes were related to disease-free survival, the detection of 13 genes makes it an inconvenience for clinical application. In contrast, our four-gene signature is more conducive to clinical use. Similar to our finding, Shen et al. adopted the method of bioinformatics to explore expression profile chip data from Gene Expression Omnibus (GEO) and the RNAseq tertiary dataset from TCGA database of HNSCC, three key genes (TGFBI, SPP1, and LAMB3) related to HNSCC prognosis were identified [55]. In another study, differentially expressed genes from TCGA were analyzed and significantly enriched in the GO and KEGG pathways of mitosis, cell cycle, Wnt, JAK/STAT, and TLR signaling pathways [56]. Liu et al. identified an eight-mRNA signature and a nomogram to predict the prognosis of HNSCC and also found that the EMT pathway plays a crucial role in their signature [57]. However, the three researches mentioned above were only based on TCGA (and GEO) dataset and have not been validated by other relevant evidence and experiments. In this study, the initial genes were obtained from a large number of published literature based on the text mining method, and then, the differences in expression and survival were verified in TCGA/GTEx database.

To further validate these candidate genes' expression and prognostic value as a whole, we conducted gene set variation analysis, showing that a high GSVA score predicts poor survival, which consists of the single gene survival analysis.

Immunotherapy plays a critical role in the treatment of HNCSS, especially in recurrent and metastatic diseases [58]. Tumor-infiltrating lymphocytes and immunological scores are linked to HNSCC prognosis and predicted radiation and chemotherapy effectiveness [59]. HNSCC is an immunosuppressive cancer that causes a broad range of immunosuppressive cells to be generated. Here, we examined the relationship between the candidate genes and immune cells and found that the infiltration of CD4+ T cell, neutrophil, macrophage, and dendritic cell was closely related to the high expression of genes. However, in HNSCC, the fundamental mechanism of immune infiltration against tumor response is still unknown. Jiang et al. argue that macrophages, T cells CD8, and T cells CD4 memory are the most commonly infiltrated subtypes of immune cells in HNSCC [60], while Wang et al. found a higher abundance of B cells, CD8+ T cells, neutrophils, and DCs in the low-risk group compared with the high-risk group, which predicts better prognosis [61]. We hypothesize that this is related to the heterogeneity of head and neck tumors and the fact that some immune cells have different subtypes that have reverse effects on prognosis.

To further validate these candidate genes' expression and prognostic value as a whole, we conducted gene set variation analysis, showing that a high GSVA score predicts poor survival, which consists of the single gene survival analysis. However, this study has certain limitations; firstly, the data on gene expression levels were obtained from public databases, and tumor specimens from patients in our hospital were not collected for verification. Secondly, the specific relationship and mechanism between these genes and radiotherapy resistance need to be further verified by experiments.

## 5. Conclusions

In this study, adopting the method of text mining and bioinformatics, we finally screened out four genes (EPHB2, SPP1, SERPINE1, and VEGFC) and corresponding signaling pathways that might be related to radiotherapy resistance in HNSCC patients. These genes are more highly expressed in tumors than in normal tissue and are associated with a poor prognosis of HNSCC. This study improves our understanding of the pathogenesis and underlying molecular mechanism of radiotherapy resistance and provides clues for further elevated radiotherapy efficacy in HNSCC patients.

## Figures and Tables

**Table 1:** The top six gene ontology enrichment terms of identified four genes (^∗^terms that do not meet the *P* value < 0.05 conditions are not shown).

Category	Term	Description	Count	Gene	*P* value
GOTERM_BP_FAT	GO:0022603	Regulation of anatomical structure morphogenesis	4	SERPINE1, SPP1, VEGFC, EPHB2	2.51*E* − 04
GOTERM_BP_FAT	GO:0051240	Positive regulation of multicellular organismal process	4	SERPINE1, SPP1, VEGFC, EPHB2	6.34*E* − 04
GOTERM_BP_FAT	GO:2000026	Regulation of multicellular organismal development	4	SERPINE1, SPP1, VEGFC, EPHB2	0.001102607
GOTERM_BP_FAT	GO:0001525	Angiogenesis	3	SERPINE1, VEGFC, EPHB2	0.001811168
GOTERM_BP_FAT	GO:0009605	Response to external stimulus	4	SERPINE1, SPP1, VEGFC, EPHB2	0.001927546
GOTERM_BP_FAT	GO:0048514	Blood vessel morphogenesis	3	SERPINE1, VEGFC, EPHB2	0.002552747
GOTERM_CC_FAT	GO:0031093	Platelet alpha granule lumen	2	SERPINE1, VEGFC	0.011315988
GOTERM_CC_FAT	GO:0031091	Platelet alpha granule	2	SERPINE1, VEGFC	0.01540963
GOTERM_CC_FAT	GO:0034774	Secretory granule lumen	2	SERPINE1, VEGFC	0.017656309
GOTERM_CC_FAT	GO:0060205	Cytoplasmic membrane-bounded vesicle lumen	2	SERPINE1, VEGFC	0.021528882
GOTERM_CC_FAT	GO:0031983	Vesicle lumen	2	SERPINE1, VEGFC	0.021732419
GOTERM_CC_FAT	GO:0005615	Extracellular space	3	SERPINE1, SPP1, VEGFC	0.027662649
GOTERM_MF_FAT	GO:0005102	Receptor binding	4	SERPINE1, SPP1, VEGFC, EPHB2	8.41*E* − 04

## Data Availability

The data used to support the findings of this study are partially included in the article. And others are available from the corresponding author upon request.

## Supplementary Materials

Supplementary Materials Figure S1: validation of the expression of genes in gene module 1 in HNSC datasets. The cutoff: ∣log_2_ fold change (FC) | ≥1, and *P* < 0.01 (∗ indicates *P* < 0.01). Figure S2: validation of the expression of genes in gene module 2 in HNSC datasets. The cutoff: ∣log_2_ fold change (FC) | ≥1, and *P* < 0.01 (∗ indicates *P* < 0.01). Figure S3: overall survival (OS) analysis of genes in gene module 1 in HNSC datasets. Figure S4: overall survival (OS) analysis of genes in gene module 2 in HNSC datasets; HNSC: head and neck squamous cell carcinoma; HR: hazard ratio; TPM: transcripts per kilobase million.
