# Supplementary figures and images for: A Four-Gene Signature Associated with Radioresistance in Head and Neck Squamous Cell Carcinoma Identified by Text Mining and Data Analysis
Source: Comput Math Methods Med. 2022 Sep 27;2022:5693806. doi: 10.1155/2022/5693806 (PMC9532131; doi:10.1155/2022/5693806)

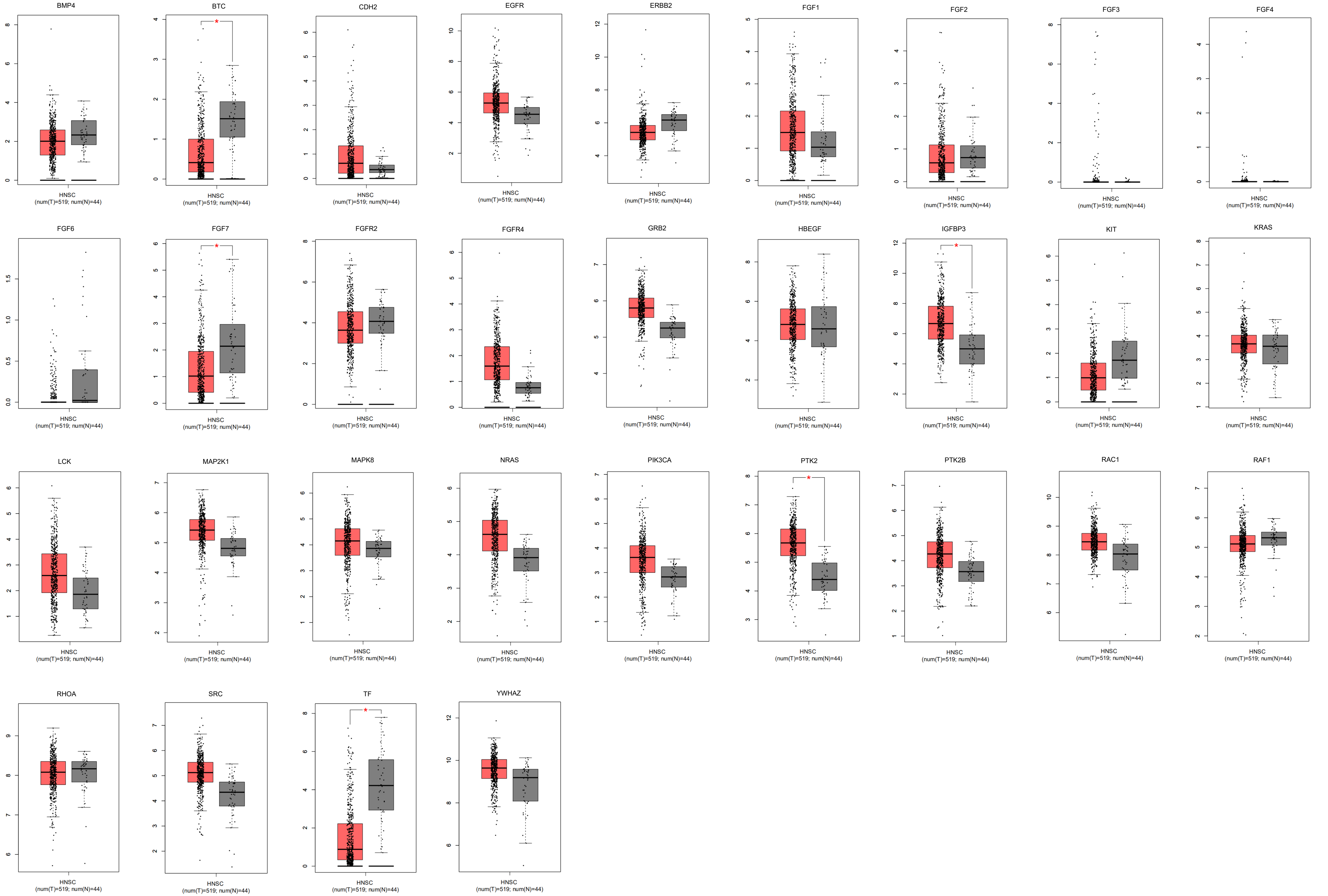

Supplement: Supplementary Materials — Figure S1: validation of the expression of genes in gene module 1 in HNSC datasets. The cutoff: ∣log2 fold change (FC) | ≥1, and P < 0.01 (∗ indicates P < 0.01). Figure S2: validation of the expression of genes in gene module 2 in HNSC datasets. The cutoff: ∣log2 fold change (FC) | ≥1, and P < 0.01 (∗ indicates P < 0.01). Figure S3: overall survival (OS) analysis of genes in gene module 1 in HNSC datasets. Figure S4: overall survival (OS) analysis of genes in gene module 2 in HNSC datasets; HNSC: head and neck squamous cell carcinoma; HR: hazard ratio; TPM: transcripts per kilobase million. [file 5693806.f1.zip › Figure S1.pdf]

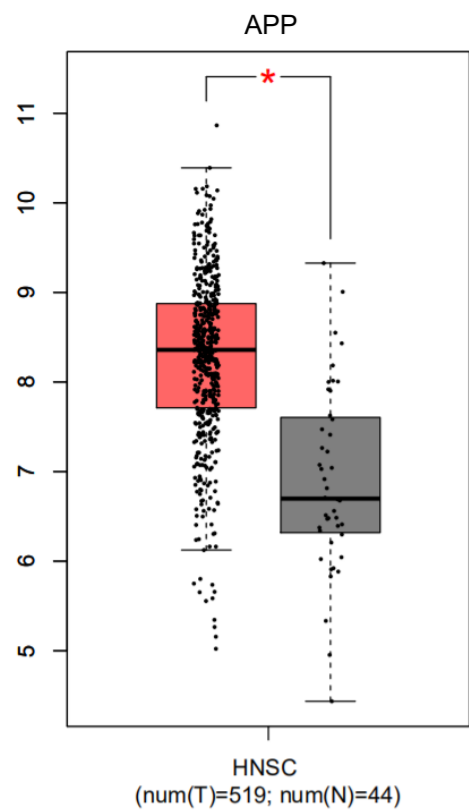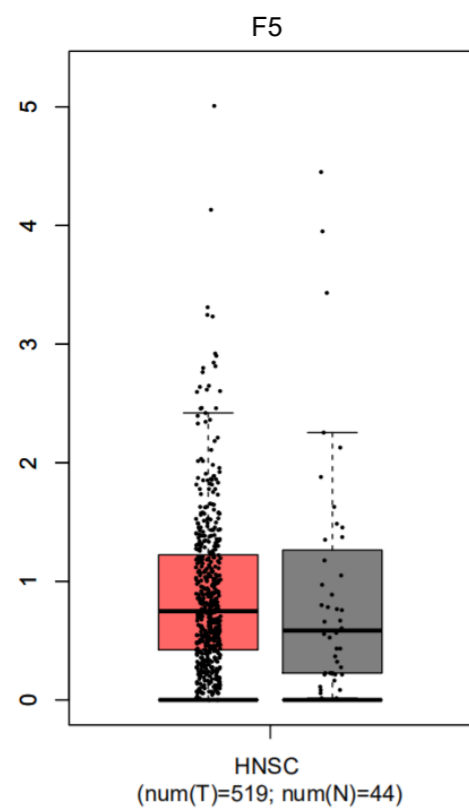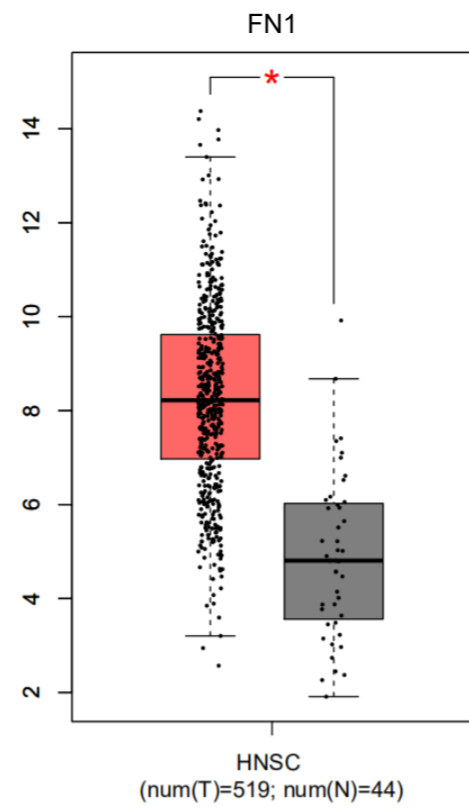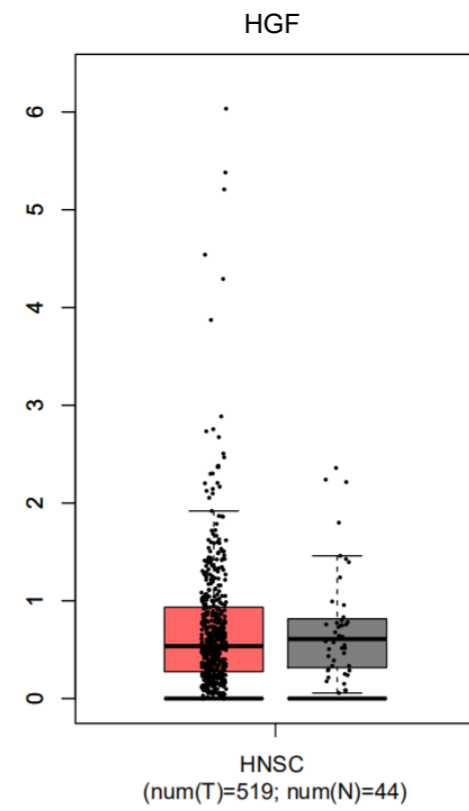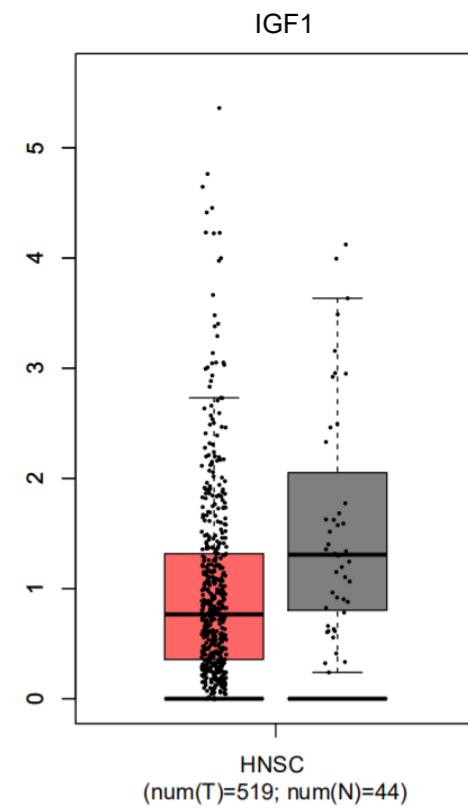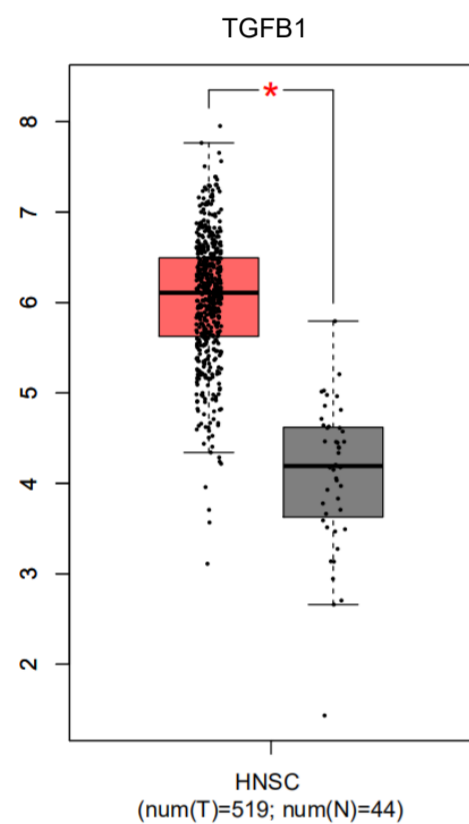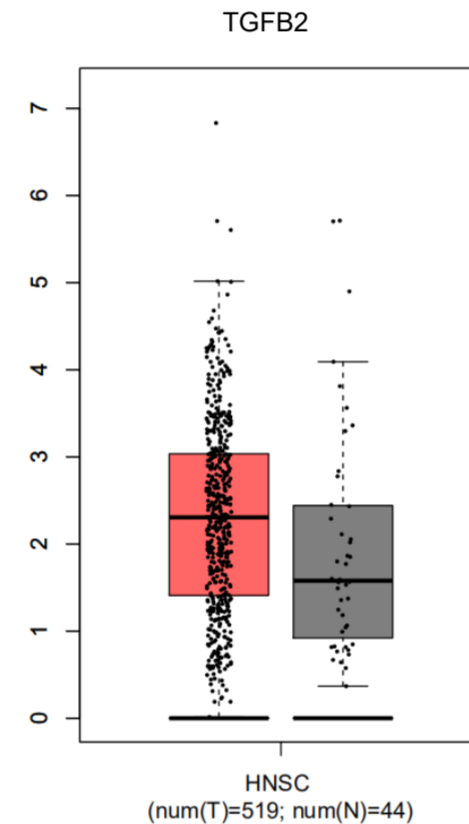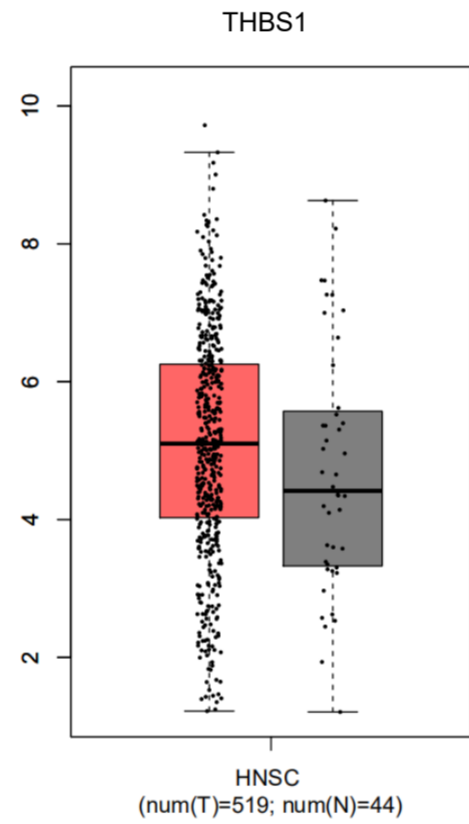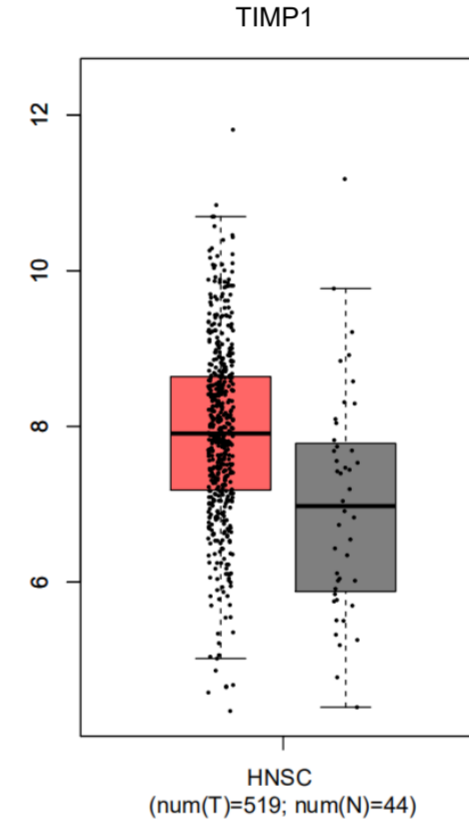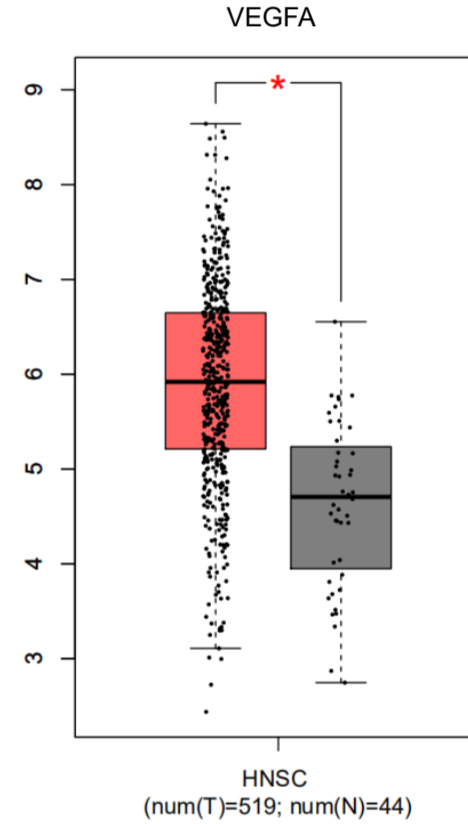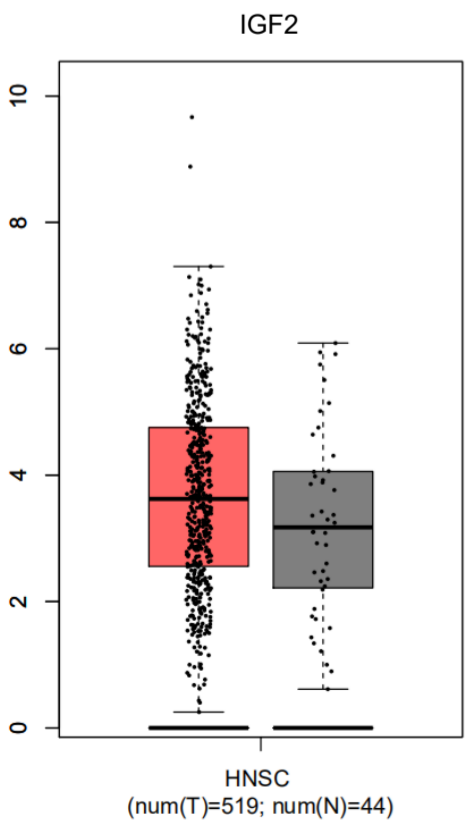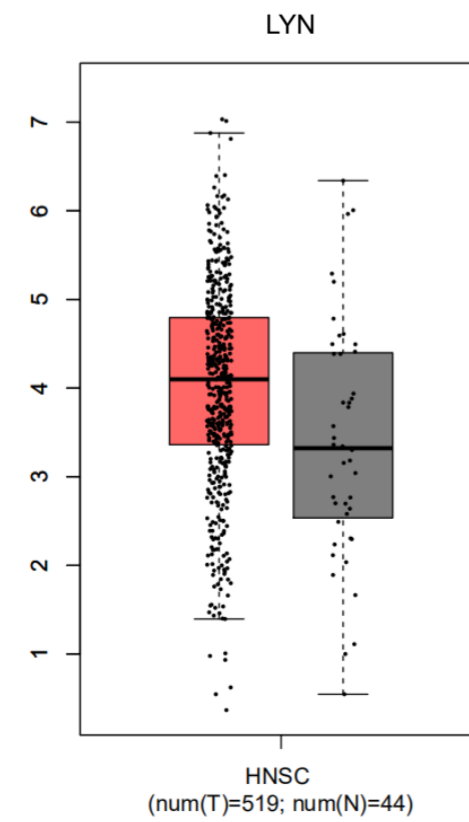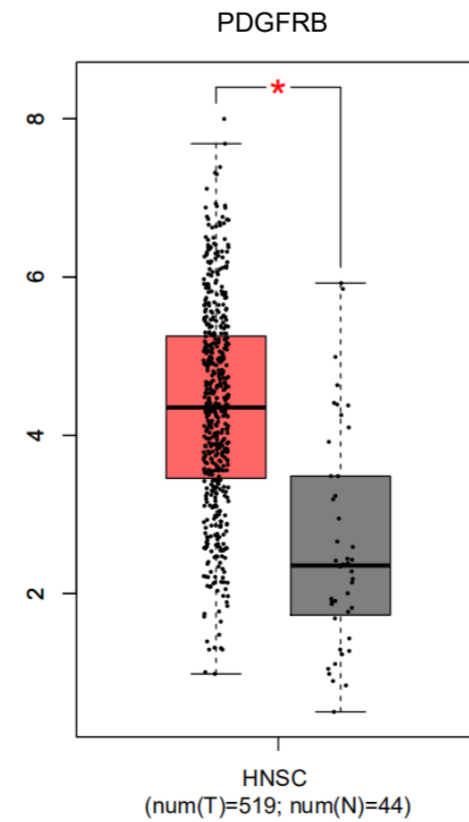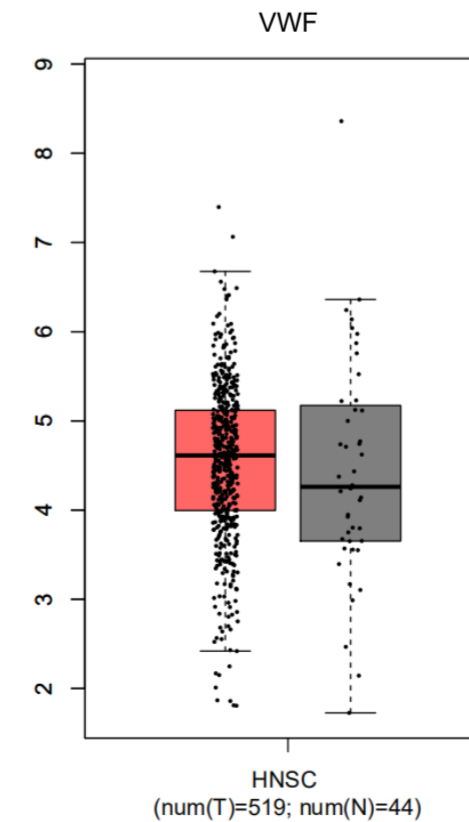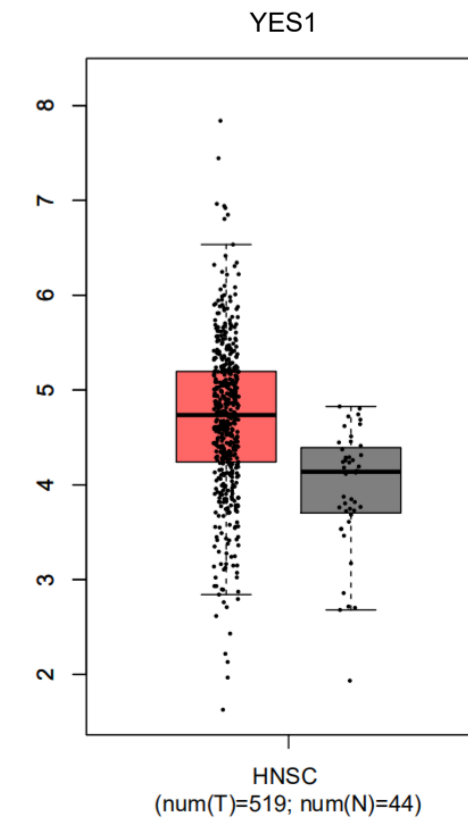

Supplement: Supplementary Materials — Figure S1: validation of the expression of genes in gene module 1 in HNSC datasets. The cutoff: ∣log2 fold change (FC) | ≥1, and P < 0.01 (∗ indicates P < 0.01). Figure S2: validation of the expression of genes in gene module 2 in HNSC datasets. The cutoff: ∣log2 fold change (FC) | ≥1, and P < 0.01 (∗ indicates P < 0.01). Figure S3: overall survival (OS) analysis of genes in gene module 1 in HNSC datasets. Figure S4: overall survival (OS) analysis of genes in gene module 2 in HNSC datasets; HNSC: head and neck squamous cell carcinoma; HR: hazard ratio; TPM: transcripts per kilobase million. [file 5693806.f1.zip › Figure S2.pdf]

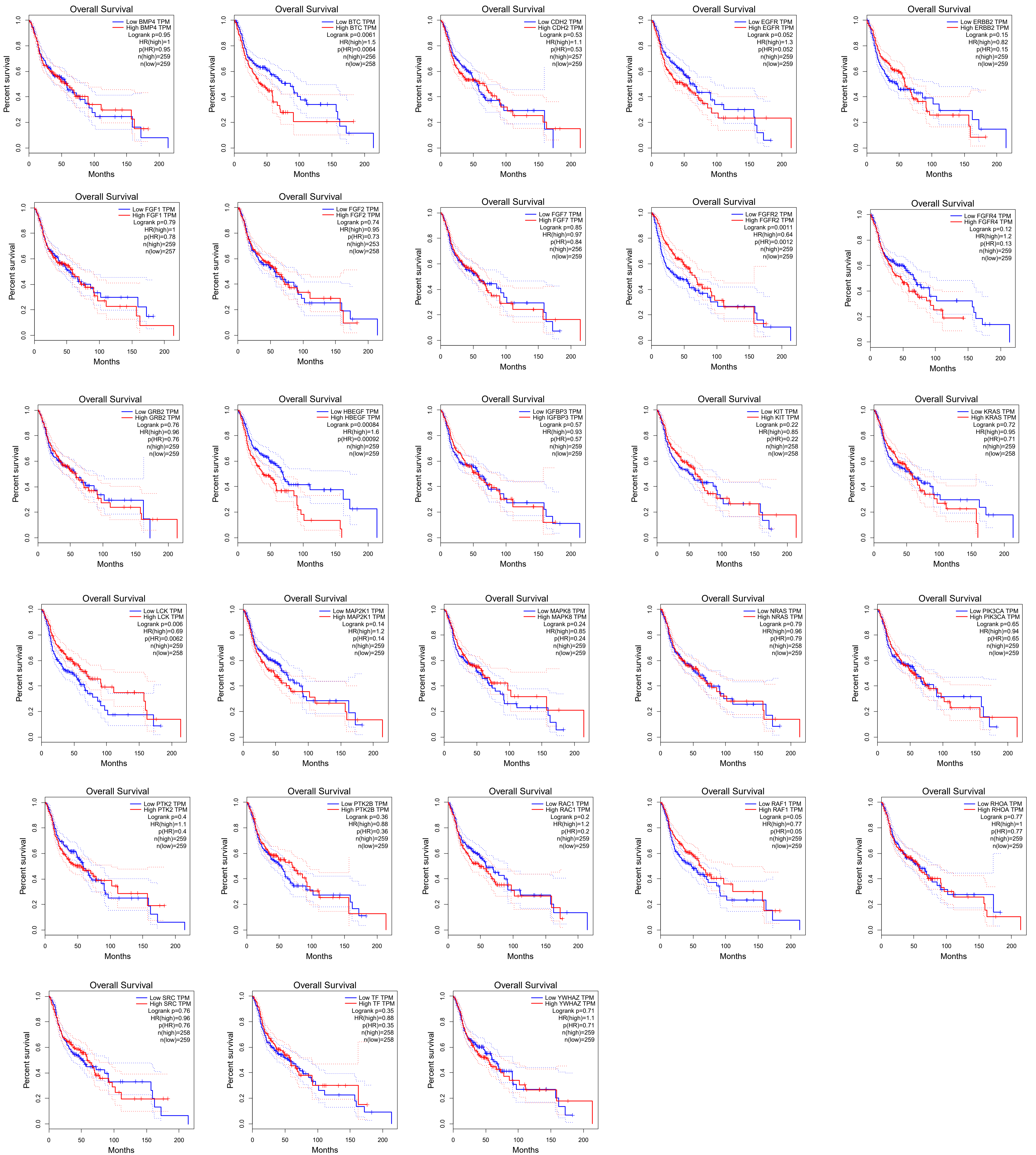

Supplement: Supplementary Materials — Figure S1: validation of the expression of genes in gene module 1 in HNSC datasets. The cutoff: ∣log2 fold change (FC) | ≥1, and P < 0.01 (∗ indicates P < 0.01). Figure S2: validation of the expression of genes in gene module 2 in HNSC datasets. The cutoff: ∣log2 fold change (FC) | ≥1, and P < 0.01 (∗ indicates P < 0.01). Figure S3: overall survival (OS) analysis of genes in gene module 1 in HNSC datasets. Figure S4: overall survival (OS) analysis of genes in gene module 2 in HNSC datasets; HNSC: head and neck squamous cell carcinoma; HR: hazard ratio; TPM: transcripts per kilobase million. [file 5693806.f1.zip › Figure S3.pdf]

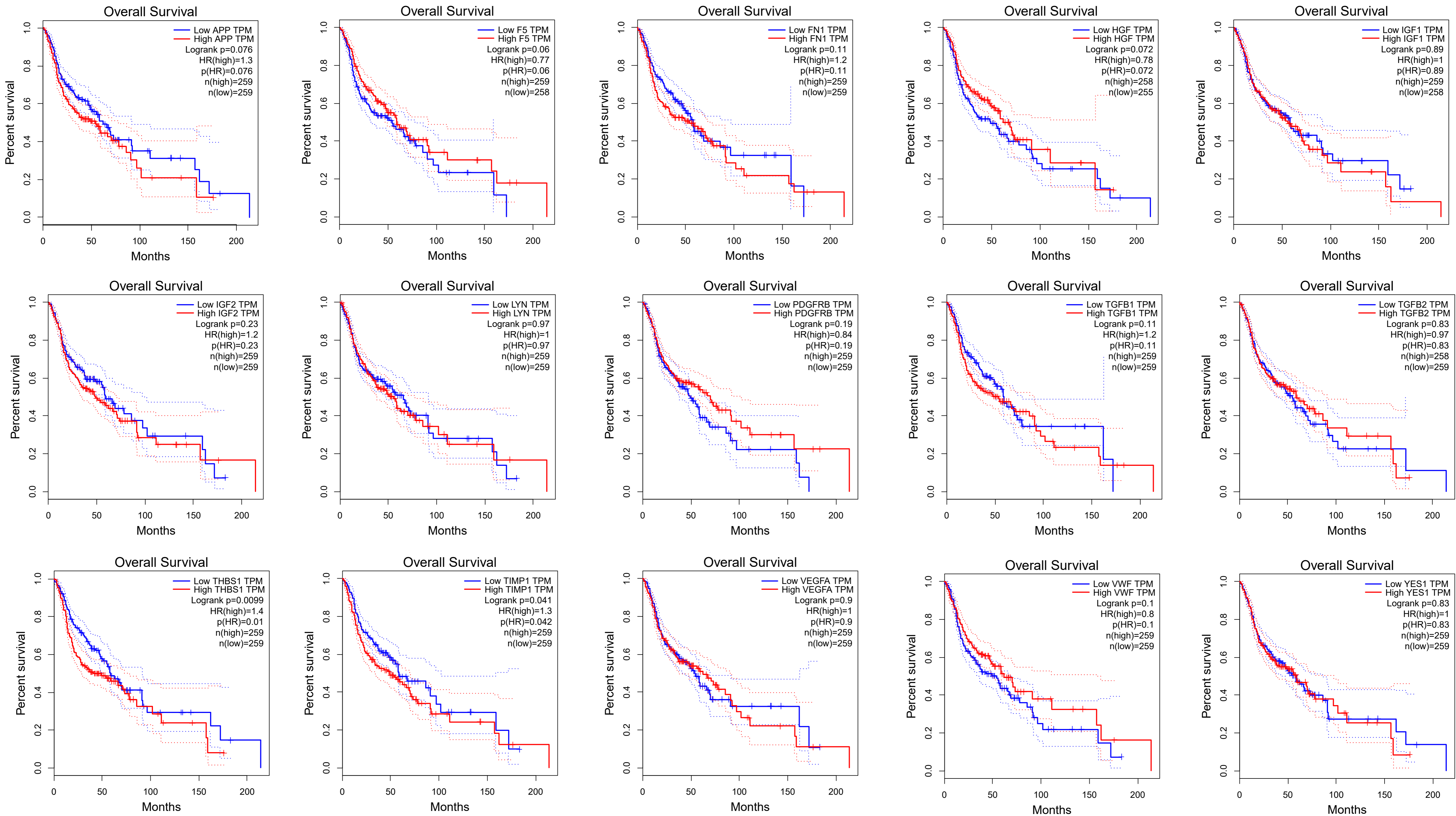

Supplement: Supplementary Materials — Figure S1: validation of the expression of genes in gene module 1 in HNSC datasets. The cutoff: ∣log2 fold change (FC) | ≥1, and P < 0.01 (∗ indicates P < 0.01). Figure S2: validation of the expression of genes in gene module 2 in HNSC datasets. The cutoff: ∣log2 fold change (FC) | ≥1, and P < 0.01 (∗ indicates P < 0.01). Figure S3: overall survival (OS) analysis of genes in gene module 1 in HNSC datasets. Figure S4: overall survival (OS) analysis of genes in gene module 2 in HNSC datasets; HNSC: head and neck squamous cell carcinoma; HR: hazard ratio; TPM: transcripts per kilobase million. [file 5693806.f1.zip › Figure S4.pdf]
